# Study on Transient Queue-Size Distribution in the Finite-Buffer Model with Batch Arrivals and Multiple Vacation Policy

**DOI:** 10.3390/e23111410

**Published:** 2021-10-27

**Authors:** Wojciech M. Kempa, Rafał Marjasz

**Affiliations:** 1 Department of Mathematics Applications and Methods for Artificial Intelligence, Faculty of Applied Mathematics, Silesian University of Technology, 23 Kaszubska Str., 44-100 Gliwice, Poland; 2 Institute of Theoretical and Applied Informatics, Polish Academy of Sciences, 5 Bałtycka Str., 44-100 Gliwice, Poland; rmarjasz@iitis.pl

**Keywords:** finite buffer, multiple vacation policy, potential method, queue size, transient state

## Abstract

The transient behavior of the finite-buffer queueing model with batch arrivals and generally distributed repeated vacations is analyzed. Such a system has potential applications in modeling the functioning of production systems, computer and telecommunication networks with energy saving mechanism based on cyclic monitoring the queue state (Internet of Things, wireless sensors networks, etc.). Identifying renewal moments in the evolution of the system and applying continuous total probability law, a system of Volterra-type integral equations for the time-dependent queue-size distribution, conditioned by the initial buffer state, is derived. A compact-form solution for the corresponding system written for Laplace transforms is obtained using an algebraic approach based on Korolyuk’s potential method. An illustrative numerical example presenting the impact of the service rate, arrival rate, initial buffer state and single vacation duration on the queue-size distribution is attached as well.

## 1. Introduction

Mathematical models of queueing systems with limited capacity of buffers accumulating customers waiting for service are widely used in practice. Among many different areas of their application, it is worth mentioning issues related to modeling the functioning of production systems, problems arising in transport issues or logistics. However, particular applications of systems with finite waiting rooms have been found in modeling the functioning of telecommunication and computer network nodes. Indeed, phenomena, such as the merging of many independent streams of incoming packets (packet queueing), packet processing (service) but also possible overflows of accumulating buffers and packet losses because of too much disproportion between the intensity of the input traffic and the speed of processing, occur there. Dynamically developing the branch of wireless communication (Wi-Fi, LTE, sensor networks, etc.) forces the search for solutions that, in addition to modeling the packet processing itself, ensure the implementation of tasks resulting from the need for energy saving. In the field of modeling the energy saving mode in wireless network nodes, queueing systems with various types of mechanisms of the periodic suspension of customer processing are used. Among many solutions, it is worth mentioning the *N*-policy threshold-type discipline, originally proposed in [1], in which the processing restarts after an idle period simultaneously with the *N*th arrival, and the *T*-policy, introduced in [2], in which the server is being activated exactly *T* time units after the last busy period. Moreover, in a fundamental paper [3], single and multiple vacation policies were proposed in which a single or repeated independent vacations are taken until at least one customer present in the buffer is detected. The results obtained in [1,3] for the M/G/1-type queue were next generalized in [4,5] for the batch Poisson arrival stream and for the M/M/c queue, respectively. An overview of results obtained by using the supplementary variable technique for non-Markovian queues with different-type server vacations can be found in [6]. In [7], the strategic joining behavior of jobs in a Markovian queueing system with Bernoulli vacation is investigated.

In the article, we consider the queueing model with multiple vacation periods launched after the completion of each busy period of the system. Queueing systems of this type can be used in the practical modeling of the functioning of, for example, computer and telecommunication network nodes (in particular wireless, e.g., based on Wi-Fi or LTE standards), in which an energy saving mechanism is implemented based on the cyclical checking the state of queue of packets waiting for processing). A multiple vacation period may also be used to perform some other operations for the service station (such as e.g., maintenance, software update, etc.). As one can observe, the literature on this type of model is extensive and constantly growing. However, as it seems, the results relate almost exclusively to the stationary state of the system. It is rare to encounter new analytical results for the transient state (at the fixed time moment *t*). In [8], a batch-arrival model with Poisson input flow, in which the processing consists of two phases, was studied. Autocorrelated input streams with models governed by the multiple vacation policy were considered in [9,10], where setup and close-down times are added into the control mechanism, and the MAP and BMAP arrival processes were assumed, respectively. In [11], a model with dependent arrival and service processes was analyzed. A BMAP-type arrival stream in a queueing system with multiple vacations and setup times was considered in [12]. An interesting modification of the classical multiple vacation scheme can be found in [13], where the model with a fixed maximal number of vacations was investigated. A queue with a control mechanism based on Active Queue Management (AQM) was considered in [14] via diffusion approximation. In [15], an infinite-buffer system of the GI/M/1 type with multiple exponentially distributed vacations was considered in the stationary state. The results obtained there were generalized for the case of multiple working vacations in [16], where the server repeatedly starts the operation at a slower speed instead of suspending it completely. Discrete-time queueing models with a multiple vacations policy were analyzed, for example, in [17,18]. A finite-buffer model of the M/G/1/N-type with vacations and exhaustive service was investigated in [19]. The problem of a stochastic decomposition structure of the busy period in a finite-buffer discrete-time vacation queue was discussed in [20]. One can find a comprehensive review of many important analytical results for different vacation queueing models, for example, in [21,22,23]. Equilibrium strategies in models with working vacations are studied in [24,25]. In [26], a Markovian queueing model with server vacations caused by breakdowns was investigated in a stationary regime. Various steady-state results for queueing models with an energy saving based on multiple vacation mechanism with additional, more complex control policies can be found, for example, in [27,28,29,30,31,32]. In fact, queueing models with a multiple vacation policy have many applications in different real-life areas, for example, in the case of customer service in a bank or at the post office, individual checkout windows are periodically closed in the event of a lack of customers: then clerks deal with other tasks (e.g., organizing documentation, preparing reports, etc.), periodically monitoring the state of the queue of customers (see e.g., [33]). An example of applying the concept of a queueing system with multiple vacations in the financial sector can be found, for example [34]. The optimal pricing server strategies under the ex-postpayment and the ex-antepayment schemes are analyzed there.

The transient analysis of the M/M/1-type system with multiple exponential vacations and additional threshold-type *N*-policy can be found in [35]. In [36], a compact-form solution for the queue-size distribution in the M/M/1-type queue with a multiple vacation policy and at most *J* single vacations are obtained. An infinite-capacity queueing system of the M/G/1 type with batch arrivals was investigated in [37,38] in the non-stationary state. Analytical results for departure process and queue-size distribution were obtained there, for the case of multiple and single vacation policies, respectively.

To the best of the authors’ knowledge, in the literature there are no analytical results in the transient state for queueing models with a multiple vacation policy, in which processing time and single vacation duration are generally distributed. In this article, we study the time-dependent queue-size distribution in the $M^{X}/G/1/N$-type queueing model with a multiple vacation policy and an exhaustive service. Applying a theoretical approach based on identifying Markovian moments in the evolution of the system, we construct a Volterra-type system of integral equations for the transient queue-size distribution conditioned by the buffer filling level at starting time t=0. For the corresponding system written for Laplace transforms (LTs for short) we use the idea of Korolyuk’s potential (see [39]) to obtain the solution in the compact form. So, the main contribution of the paper is the closed-form representation for the LT of the conditional queue-size distribution in the non-stationary state of the system. The formula is written by means of a certain functional sequence (in [39] called a potential) with terms, which can be obtained recursively or with the help of power expansion. Numerical examples illustrate analytical results, namely the impact of the vacation duration, arrival intensity, service speed, buffer size and the initial system state on the behavior of the queue-size distribution.

The remaining part of the article is organized as follows. In Section 2, we provide a detailed description of the considered queueing model and state the necessary nomenclature. In Section 3, we obtain the system of integral equations for the conditional queue-size distribution in the transient state. In Section 4, we sketch the idea of Korolyuk’s potential approach and use it to obtain the compact-form solution of the corresponding system written for LTs. Section 5 is devoted to numerical computations illustrating theoretical results. To visualize the appropriate probabilities we use one of the algorithms of numerical LT inversion. Moreover, comparing numerical results to the simulation is conducted. Finally, Section 6 contains a summary and short conclusions.

## 2. Mathematical Description of the Model

We deal with the $M^{X}/G/1/N$-type queueing model in which groups (batches) of packets arrive according to a compound Poisson process with intensity λ. Group sizes are generally-distributed random variables and $p_{k}$ stands for the probability that the arriving group consists of *k* packets exactly, where $\sum_{k=1}^{\infty }p_{k}=1.$ The processing is organized according to the natural FIFO discipline and the service time of an individual packet is a random variable with a general-type cumulative distribution function (CDF for short) F(·). The system capacity equals N,, that is, there is an accumulating buffer with N−1 places for waiting packets and one place reserved for the packet being processed.

The considered queueing system is governed by the multiple vacation policy. Namely, every time the server becomes idle (at the completion epoch of each busy period) the server takes a vacation of a random duration with a general CDF G(·). After the vacation, the buffer state is examined. If there are no packets waiting for service, the next independent vacation is started and so on. The last vacation is the one at the completion epoch of which at least one packet waiting for processing is detected. Successive server vacations started as long as the buffer is empty are called a multiple vacation period (MVP for short). So, the operation of the system can be observed in successive busy periods, during which the processing of packets is uninterrupted, followed by MVPs during which the processing is suspended.

We end this section by introducing necessary notations. We denote by f(·) and g(·) Laplace–Stieltjes transforms (LST for short) of CDFs F(·) and G(·), respectively. Similarly, tails of CDFs F(·) and G(·) are denoted by $\bar{F}\left(\cdot \right)$ and $\bar{G}\left(\cdot \right),$ respectively. We define the *i*-fold Stieltjes transform of G(·) with itself as follows:

(1) $$\begin{array}{cc} & G^{0*}\left(t\right)=1,\;G^{i*}\left(t\right)=\int_{0}^{t}G^{(i-1)*}\left(t-y\right)dG\left(y\right), \end{array}$$

where t>0 and i≥1.

Similarly, $p_{j}^{i*}$ stands for the *j*th term of the *i*-fold convolution of the sequence $\left(p_{k}\right)$ with itself, namely:

(2) $$\begin{array}{cc} & p_{j}^{0*}=I\left\{j=0\right\},\;p_{j}^{i*}=\sum_{k=1}^{j}p_{k}^{(i-1)*}p_{j-k}, \end{array}$$

where i≥1, and I{·} is the indicator defined as:

(3) $$\begin{array}{cc} & I\left\{E\right\}=\left\{\begin{array}{cc} 1,\; & \mathrm{if}\;\mathrm{the}\;\mathrm{event}\;\;E\;\;\mathrm{occurs},\\ 0,\; & \mathrm{otherwise}. \end{array}\right. \end{array}$$

Finally, let us denote by X(t) the number of packets present in the system at time *t* (including the one being processed at this time, if any).

## 3. Equations for Conditional Queue-Size Distribution

In this section, identifying Markovian moments in the evolution of the system, we build a system of integral equations for the transient queue-size distribution conditioned by the buffer state at the starting time t=0.

We introduce the following notation:

(4) $$\begin{array}{cc} & P_{n}\left(t,m\right)\overset{def}{=}P\left\{X\left(t\right)=m\;|\;X\left(0\right)=n\right\}, \end{array}$$

where t>0 and 0≤m,n≤N.

Let us consider firstly the case of the system being empty at the starting epoch t=0. In such a case we assume that an MVP begins at this moment, so we identify the starting time t=0 with the completion of a busy period. Note that the continuous total probability law leads to the following integral equation (having in mind that λ>0):

(5) $$\begin{array}{cc} P_{0}\left(t,m\right)= & \sum_{i=0}^{\infty }\int_{u=0}^{t}dG^{i*}\left(u\right)\int_{y=u}^{t}\lambda e^{-\lambda y}dy\int_{v=y-u}^{t-u}\\ & [\sum_{k=1}^{N-1}p_{k}\sum_{j=0}^{N-k-1}\sum_{r=0}^{j}p_{j}^{r*}\frac{{\left[\lambda (u+v-y)\right]}^{r}}{r!}e^{-\lambda (u+v-y)}P_{k+j}\left(t-u-v,m\right)\\ & +P_{N}\left(t-u-v,m\right)\left(\sum_{k=N}^{\infty }p_{k}+\sum_{k=1}^{N-1}p_{k}\sum_{j=N-k}^{\infty }\sum_{r=0}^{j}p_{j}^{r*}\frac{{\left[\lambda (u+v-y)\right]}^{r}}{r!}e^{-\lambda (u+v-y)}\right)]dG\left(v\right)\\ & +\sum_{i=0}^{\infty }\int_{u=0}^{t}dG^{i*}\left(u\right)\int_{y=u}^{t}\lambda e^{-\lambda y}\bar{G}\left(t-u\right)dy\\ & \cdot [I\left\{1\leq m\leq N-1\right\}\sum_{k=1}^{m}p_{k}\sum_{j=0}^{m-k}p_{m-k}^{j*}\frac{{\left[\lambda (t-y)\right]}^{j}}{j!}e^{-\lambda (t-y)}\\ & +I\left\{m=N\right\}\left(\sum_{k=N}^{\infty }p_{k}+\sum_{k=1}^{N-1}p_{k}\sum_{j=N-k}^{\infty }\sum_{r=0}^{j}p_{j}^{r*}\frac{{\left[\lambda (t-y)\right]}^{r}}{r!}e^{-\lambda (t-y)}\right)]+I\left\{m=0\right\}e^{-\lambda t}. \end{array}$$

Let us comment briefly on the above formula. The moment of the first packet arrival is denoted by y, while the last single vacation before time *t* (distributed with a CDF G(·)) completes at time u. Two components in the first square brackets on the right side of (5) correspond to the situation where the first MVP completes before time *t* (the completion epoch of the MVP is denoted by u+v). The first summand relates to the case in which there is at least one place free in the accumulating buffer at time u+v (this moment also coincides with the starting moment of the busy period). So, if k≤N−1 packets enter at time y, then next, at most N−k−1 packets arrive during a time period of length u+v−y. The second summand describes the case of the buffer being saturated at the starting time of the busy period. The second square brackets on the right side of (5) represent the situation in which the MVP ends after time t, however, at least one packet arrives before t. If m≤N−1 then the number of arrivals up to the moment *t* equals *m* exactly (the first summand). In the case m=N, the number of arrivals must be greater than or equal to *N* (the second summand). Finally, the expression $I\left\{m=0\right\}e^{-\lambda t}$ corresponds to the case of no arrivals before time t. In this case, X(t)=m if and only if m=0.

Consider now the case where the buffer contains at least one packet at the starting moment t=0. As it is well known, $(X\left(t_{n}+\right)),$ where $t_{1}<t_{2}<\ldots ,$ are successive service completion (departure) epochs, is an embedded Markov chain (EMC) in the $M^{X}/G/1$-type queue. The continuous version of the total probability law applied with respect to the first departure epoch after the starting moment t=0 leads to the following system of integral equations:

(6) $$\begin{array}{cc} P_{n}\left(t,m\right)= & \int_{0}^{t}[\sum_{k=0}^{N-n-1}\sum_{r=0}^{k}p_{k}^{r*}\frac{{\left(\lambda y\right)}^{r}}{r!}e^{-\lambda y}P_{n+k-1}\left(t-y,m\right)\\ & +P_{N-1}\left(t-y,m\right)\sum_{k=N-n}^{\infty }\sum_{r=0}^{k}p_{k}^{r*}\frac{{\left(\lambda y\right)}^{r}}{r!}e^{-\lambda y}]dF\left(y\right)\\ & +\left(I\left\{n\leq m\leq N-1\right\}\sum_{r=0}^{m-n}p_{m-n}^{r*}\frac{{\left(\lambda t\right)}^{r}}{r!}+I\left\{m=N\right\}\sum_{k=N-n}^{\infty }\sum_{r=0}^{k}p_{k}^{r*}\frac{{\left(\lambda t\right)}^{r}}{r!}\right)e^{-\lambda t}\bar{F}\left(t\right), \end{array}$$

where λ>0 stands for the group arrival intensity and n∈{1,…,N}.

Indeed, the integral on the right side of (6) relates to the situation in which the first packet leaves the system at time y<t: the first summand in brackets corresponds to the case where there is at least one place before that moment in the accumulating buffer; in the second summand, the buffer is saturated just before the first departure, so after the first service completion epoch the state of the system equals N−1. The other two components after the integral on the right side of (6) represent the situation in which the first service ends after time t.

Let us introduce the LT of $P_{n}\left(t,m\right)$ as:

(7) P^n(s,m)=def∫0∞e−stPn(t,m)dt,Re(s)>0,

and define for s>0 the following functional sequences:

(8) $$\begin{array}{cc} \alpha_{k}\left(s\right)\overset{def}{=} & \int_{0}^{\infty }e^{-(s+\lambda )y}\sum_{r=0}^{k}p_{k}^{r*}\frac{{\left(\lambda y\right)}^{r}}{r!}dF\left(y\right), \end{array}$$

(9) $$\begin{array}{cc} \beta_{k}\left(s\right)\overset{def}{=} & \frac{1}{1-g(s+\lambda )}\int_{0}^{\infty }e^{-(s+\lambda )y}\sum_{r=0}^{k}p_{k}^{r*}\frac{{\left(\lambda y\right)}^{r+1}}{(r+1)!}dG\left(y\right), \end{array}$$

(10) $$\begin{array}{cc} \gamma \left(s\right)\overset{def}{=} & \frac{g(s)-g(s+\lambda )}{1-g(s+\lambda )}\sum_{k=N}^{\infty }p_{k}, \end{array}$$

(11) $$\begin{array}{cc} \delta \left(s,m\right)\overset{def}{=} & \frac{1}{1-g(s+\lambda )}[I\left\{1\leq m\leq N-1\right\}\sum_{k=1}^{m}p_{k}H^{\left(G\right)}\left(m-k,s\right)\\ & +I\left\{m=N\right\}\left(\sum_{k=N}^{\infty }p_{k}H^{\left(G\right)}\left(s\right)+\sum_{k=1}^{N-1}p_{k}\sum_{j=N-k}^{\infty }H^{\left(G\right)}\left(j,s\right)\right)] \end{array}$$

and

(12) $$\begin{array}{cc} \epsilon_{k}\left(s,m\right)\overset{def}{=} & I\left\{n\leq m\leq N-1\right\}H^{\left(F\right)}\left(m-k,s\right)+I\left\{m=N\right\}\sum_{k=N-k}^{\infty }H^{\left(F\right)}\left(k,s\right), \end{array}$$

where

(13) $$\begin{array}{cc} H^{\left(G\right)}\left(k,s\right)\overset{def}{=} & \int_{0}^{\infty }e^{-(s+\lambda )t}\sum_{r=0}^{k}p_{k}^{r*}\frac{{\left(\lambda t\right)}^{r+1}}{(r+1)!}\bar{G}\left(t\right)dt, \end{array}$$

(14) $$\begin{array}{cc} H^{\left(F\right)}\left(k,s\right)\overset{def}{=} & \int_{0}^{\infty }e^{-(s+\lambda )t}\sum_{r=0}^{k}p_{k}^{r*}\frac{{\left(\lambda t\right)}^{r}}{r!}\bar{F}\left(t\right)dt, \end{array}$$

(15) $$\begin{array}{cc} H^{\left(G\right)}\left(s\right)\overset{def}{=} & \int_{0}^{\infty }\left[e^{-st}-e^{-(s+\lambda )t}\right]\bar{G}\left(t\right)dt. \end{array}$$

Note that the system (5)–(6) leads now to the following corresponding one written for LTs:

(16) $$\begin{array}{cc} {\hat{P}}_{0}\left(s,m\right)= & \sum_{k=1}^{N-1}p_{k}\left[\sum_{j=0}^{N-k-1}\beta_{j}\left(s\right){\hat{P}}_{k+j}\left(s,m\right)+{\hat{P}}_{N}\left(s,m\right)\sum_{j=N-k}^{\infty }\beta_{j}\left(s\right)\right]\\ & +{\hat{P}}_{N}\left(s,m\right)\gamma \left(s\right)+\delta \left(s,m\right)+\frac{I\{m=0\}}{s+\lambda } \end{array}$$

and

(17) $$\begin{array}{cc} & {\hat{P}}_{n}\left(s,m\right)=\sum_{k=0}^{N-n-1}\alpha_{k}\left(s\right){\hat{P}}_{n+k-1}\left(s,m\right)+{\hat{P}}_{N-1}\left(s,m\right)\sum_{k=N-n}^{\infty }\alpha_{k}\left(s\right)+\epsilon_{n}\left(s,m\right), \end{array}$$

where n∈{1,…,N}.

We will now rewrite the last system of Equations (16) and (17) in a specific form that will be relevant in the context of the method that we will use to obtain the solution.

By introducing the following notation:

(18) $$\begin{array}{cc} & {\hat{Q}}_{n}\left(s,m\right)\overset{def}{=}{\hat{P}}_{N-n}\left(s,m\right),\;n\in \left\{0,\ldots ,N\right\}, \end{array}$$

Equation (17) will take the form:

(19) $$\begin{array}{cc} & \sum_{k=-1}^{n}\alpha_{k+1}\left(s\right){\hat{Q}}_{n-k}\left(s,m\right)-{\hat{Q}}_{n}\left(s,m\right)=\psi_{n}\left(s,m\right), \end{array}$$

where n∈{0,…,N−1} and

(20) $$\begin{array}{cc} \psi_{n}\left(s,m\right)\overset{def}{=} & \alpha_{n+1}\left(s\right){\hat{Q}}_{0}\left(s,m\right)-{\hat{Q}}_{1}\left(s,m\right)\sum_{k=n+1}^{\infty }\alpha_{k}\left(s\right)-\epsilon_{N-n}\left(s,m\right). \end{array}$$

Similarly, Equation (16) can be rewritten as:

(21) $$\begin{array}{cc} {\hat{Q}}_{N}\left(s,m\right)= & \sum_{k=1}^{N-1}p_{k}\left[\sum_{j=0}^{N-k-1}\beta_{j}\left(s\right){\hat{Q}}_{N-k-j}\left(s,m\right)+{\hat{Q}}_{0}\left(s,m\right)\sum_{j=N-k}^{\infty }\beta_{j}\left(s\right)\right]\\ & +{\hat{Q}}_{0}\left(s,m\right)\gamma \left(s\right)+\delta \left(s,m\right)+\frac{I\{m=0\}}{s+\lambda }. \end{array}$$

## 4. Main Analytical Result

In this section, we use Korolyuk’s potential approach to obtain the solution of the system (19) and (21) in a compact form. In [39], the following linear system with an infinite number of equations is considered:

(22) $$\begin{array}{cc} & \sum_{k=-1}^{n}\alpha_{k+1}q_{n-k}-q_{n}=\psi_{n},\;n\geq 0, \end{array}$$

where $\left(\alpha_{n}\right)$ and $\left(\psi_{n}\right)$ are two known number sequences, and $\left(q_{n}\right)$ is the sequence of unknowns. Such a system has infinitely many solutions; however, as was proved in [39], each solution of (22) can be written as follows:

(23) $$\begin{array}{cc} & q_{n}=LR_{n}+\sum_{k=1}^{n}R_{n-k}\psi_{k},\;n\geq 1, \end{array}$$

where L∈R and, in general, it can be different for different solutions, and the sequence $(R_{k}),$ called a potential in [39], is defined by the sequence of coefficients $\left(\alpha_{n}\right)$ in two equivalent ways. Firstly, its successive terms can be obtained recursively, one by one, in the following way:

(24) $$\begin{array}{cc} & R_{0}=0,\;R_{1}=\frac{1}{\alpha_{0}},\;R_{k}=R_{1}\left(R_{k-1}-\sum_{j=0}^{k-1}\alpha_{j+1}R_{k-1-i}\right), \end{array}$$

where k≥2. Alternatively, successive terms of the sequence $\left(R_{k}\right)$ can be obtained by means of power expansion. Indeed, defining:

(25) $$\begin{array}{cc} & \Omega \left(z\right)\overset{def}{=}\sum_{k=1}^{\infty }\alpha_{k}z^{k},\;\left|z\right|<1, \end{array}$$

one can easily show that

(26) $$\begin{array}{cc} & \sum_{k=0}^{\infty }R_{k}z^{k}=\frac{z}{\Omega (z)-z}. \end{array}$$

In consequence, expanding the right side of (26) in the Maclaurin series and next comparing coefficients at successive powers of *z* on the left and right sides of (26), we obtain:

(27) $$\begin{array}{cc} & R_{k}=\frac{1}{k!}\frac{d^{k}}{dz^{k}}\left(\frac{z}{\Omega (z)-z}\right){|}_{z=0}. \end{array}$$

Note that the systems of Equations (19) and (22) have the same form. However, there are two main differences. Firstly, functions in the system (22) depend on some arguments: we have $\alpha_{k}=\alpha_{k}\left(s\right),$$q_{k}=q_{k}\left(s,m\right)$ and $\psi_{n}=\psi_{n}\left(s,m\right).$ Secondly, the number of equations in (19) compared to (22) is finite. To obtain the representation for the solution of (19), we can use the result (23) assuming now that L=L(s,m) and $R_{k}=R_{k}\left(s\right).$ As it turns out, Equation (21) written for n=N will, however, serve as the boundary condition allowing for the explicit determination of L(s,m).

We prove the following main theorem:

**Theorem** **1.** *In the $M^{X}/G/1/N$-type queueing model with multiple vacation policy the representation for the Laplace transform ${\hat{P}}_{n}\left(s,m\right)$ of the conditional transient queue-size distribution is the following:*

(28) $$\begin{array}{cc} {\hat{P}}_{n}\left(s,m\right)= & \chi_{N-n}\left(s,m\right)+\Lambda_{N-n}\left(s\right)(\sum_{k=1}^{N-1}p_{k}\sum_{j=1}^{N-k}\beta_{N-k-j}\left(s\right)\chi_{j}\left(s,m\right)\\ & +\delta \left(s,m\right)+\frac{I\{m=0\}}{s+\lambda }-\chi_{N}\left(s,m\right))\\ & \cdot {\left(\Lambda_{N}\left(s\right)-\gamma \left(s\right)-\sum_{k=1}^{N-1}p_{k}\left(\sum_{j=1}^{N-k}\beta_{N-k-j}\left(s\right)\Lambda_{j}\left(s\right)+\sum_{j=N-k}^{\infty }\beta_{j}\left(s\right)\right)\right)}^{-1}, \end{array}$$

*where*

(29) $$\begin{array}{cc} \Lambda_{n}\left(s\right)= & \alpha_{0}\left(s\right)R_{n+1}\left(s\right)+\sum_{k=0}^{n}R_{n-k}\left(s\right)\left(\alpha_{k+1}\left(s\right)-\frac{1}{f(s)}\sum_{i=k+1}^{\infty }\alpha_{i}\left(s\right)\right) \end{array}$$

*and*

(30) $$\begin{array}{cc} \chi_{n}\left(s,m\right)= & \sum_{k=0}^{n}R_{n-k}\left(s\right)\left(\frac{\epsilon_{N}\left(s,m\right)}{f(s)}\sum_{i=k+1}^{\infty }\alpha_{i}\left(s\right)-\epsilon_{N-k}\left(s,m\right)\right), \end{array}$$

*the formulae for $\alpha_{k}\left(s\right),$$\beta_{k}\left(s\right),$γ(s),δ(s,m),$\epsilon_{k}\left(s,m\right)$ are given in (8), (9), (10), (11) and (12), respectively, and the sequence $\left(R_{k}\left(s\right)\right)$ is defined recursively as follows (compare (24)):*

(31) $$\begin{array}{cc} & R_{0}\left(s\right)=0,\;R_{1}\left(s\right)=\frac{1}{\alpha_{0}\left(s\right)},\;R_{k}\left(s\right)=R_{1}\left(s\right)\left(R_{k-1}\left(s\right)-\sum_{j=0}^{k-1}\alpha_{j+1}\left(s\right)R_{k-1-i}\left(s\right)\right), \end{array}$$

*where k≥1.*

**Proof.**  Obviously, since (19) has the same form as (22), then each solution of the system (19), (21) can be written in the following way (compare (23)):

(32) $$\begin{array}{cc} & {\hat{Q}}_{n}\left(s,m\right)=L\left(s,m\right)R_{n+1}\left(s\right)+\sum_{k=0}^{n}R_{n-k}\left(s\right)\psi_{k}\left(s,m\right), \end{array}$$

where n∈{0,…,N} and the sequence $\left(R_{k}\left(s\right)\right)$ is defined in (31).

To obtain the explicit representation for ${\hat{Q}}_{n}\left(s,m\right)$ we need the formula for L(s,m). Taking n=0 in (32), we have:

(33) $$\begin{array}{cc} & L\left(s,m\right)=\alpha_{0}\left(s\right){\hat{Q}}_{0}\left(s,m\right). \end{array}$$

Similarly, substituting n=0 into (19), we get:

(34) $$\begin{array}{cc} & \alpha_{0}\left(s\right){\hat{Q}}_{1}\left(s,m\right)+\alpha_{1}\left(s\right){\hat{Q}}_{0}\left(s,m\right)-{\hat{Q}}_{0}\left(s,m\right)=\psi_{0}\left(s,m\right), \end{array}$$

where (see (20))

$$\begin{array}{cc} \psi_{0}\left(s,m\right)= & \alpha_{1}\left(s\right){\hat{Q}}_{0}\left(s,m\right)-{\hat{Q}}_{1}\left(s,m\right)\sum_{k=1}^{\infty }\alpha_{k}\left(s\right)-\epsilon_{N}\left(s,m\right). \end{array}$$

Due to the fact that $\sum_{k=0}^{\infty }\alpha_{k}\left(s\right)=f\left(s\right)$ we have from (34)

(35) $$\begin{array}{cc} & {\hat{Q}}_{1}\left(s,m\right)=\frac{{\hat{Q}}_{0}\left(s,m\right)-\epsilon_{N}\left(s,m\right)}{f(s)}. \end{array}$$

Using now (33) and (35) in (32), we obtain

(36) $$\begin{array}{cc} {\hat{Q}}_{n}\left(s,m\right) & =\alpha_{0}\left(s\right)R_{n+1}\left(s\right){\hat{Q}}_{0}\left(s,m\right)+\sum_{k=0}^{n}R_{n-k}\left(s\right)\left[\alpha_{k+1}\left(s\right){\hat{Q}}_{0}\left(s,m\right)-{\hat{Q}}_{1}\left(s,m\right)\sum_{i=k+1}^{\infty }\alpha_{i}\left(s\right)-\epsilon_{N-k}\left(s,m\right)\right]\\ & =\Lambda_{n}\left(s\right){\hat{Q}}_{0}\left(s,m\right)+\chi_{n}\left(s,m\right), \end{array}$$

where functions $\Lambda_{n}\left(s\right)$ and $\chi_{n}\left(s,m\right)$ are defined in (29) and (30), respectively.

Now let us use Equation (21) as a specific boundary condition in order to find the formulae for ${\hat{Q}}_{0}\left(s,m\right).$ Applying (36) in (21) gives:

(37) $$\begin{array}{cc} {\hat{Q}}_{0}\left(s,m\right)= & \left(\sum_{k=1}^{N-1}p_{k}\sum_{j=1}^{N-k}\beta_{N-k-j}\left(s\right)\chi_{j}\left(s,m\right)+\delta \left(s,m\right)+\frac{I\{m=0\}}{s+\lambda }-\chi_{N}\left(s,m\right)\right)\\ & \cdot {\left(\Lambda_{N}\left(s\right)-\gamma \left(s\right)-\sum_{k=1}^{N-1}p_{k}\left(\sum_{j=1}^{N-k}\beta_{N-k-j}\left(s\right)\Lambda_{j}\left(s\right)+\sum_{j=N-k}^{\infty }\beta_{j}\left(s\right)\right)\right)}^{-1}. \end{array}$$

Now, referring to (18) and (36), completes the proof. □

## 5. Numerical Study

In this section, we investigate numerically the impact of the main “input” system parameters (like arrival intensity, service rate or mean vacation duration) on the queue-size distribution. To obtain the transient queue-size distribution from the formula (28) for particular system parameters we use the Gaver–Stehfest algorithm of the numerical Laplace transform inversion described in detail in [40,41] (see also [42,43]). In computations, we deal with a model described as follows:

- Poisson arrivals of packets of sizes 100 B;
- CDF of the processing time is a mixture of two exponential distributions (second-order hyper-exponential distribution) and is defined as:
  $$\begin{array}{cc} & F\left(t\right)=\alpha \cdot \left(1-e^{-\mu_{1}t}\right)+\left(1-\alpha \right)\cdot \left(1-e^{-\mu_{2}t}\right),\;t>0, \end{array}$$
  where $\alpha ,\mu_{1}$ and $\mu_{2}$ are given;
- CDF of a single server vacation has 2-Erlang distribution with fixed parameter ξ, namely:
  $$\begin{array}{cc} & G\left(t\right)=1-e^{-\xi t}\left(1+\xi t\right),\;t>0; \end{array}$$
- buffer size equals N=10 packets (so 1 kB).

A hyper-exponential probability distribution of single packet’s processing time can be used in modeling the situation in which a single packet (customer, task, message, etc.) can receive two types of service: normal (typical) and special, for which the average duration times differ significantly. The value of parameter α indicates the fraction of incoming packets, which receive typical service that takes on average $\mu_{1}^{-1}$ time units. The other packets are processed according to a special mode.

Since during the server idle mode (multiple vacation period) the service station can perform other tasks (such as e.g., background tasks of the CPU processor), the 2-Erlang distribution can be used to approximate a single vacation period duration: we can assume that it consists of two consecutive subperiods of random length. During the first of them, the task is prepared to run in the background, and its direct execution takes place in the second period.

The result obtained in Theorem 1 is of a general nature (i.e., CDFs F(·),G(·) and the sequence $\left(p_{k}\right)$ are general). However, in numerical computations we use the fact that the right side of (28) is a rational function of variable s. If ${\hat{P}}_{n}\left(s,m\right)$ is not a rational function of *s* we can use, for example, the classical technique of Padé approximation of a given Laplace transform by an appropriate rational function (see, e.g., [44]).

### 5.1. Impact of Service Rate

Let us investigate firstly the impact of the service rate. Consider three scenarios in which the parameters of the service time CDF are α=0.4 and

$$\begin{array}{cc} & \left(\mu_{1},\mu_{2}\right)\in \left\{\left(960,720\right);\left(780,520\right);\left(320,720\right)\right\}. \end{array}$$

**Figure 1:**
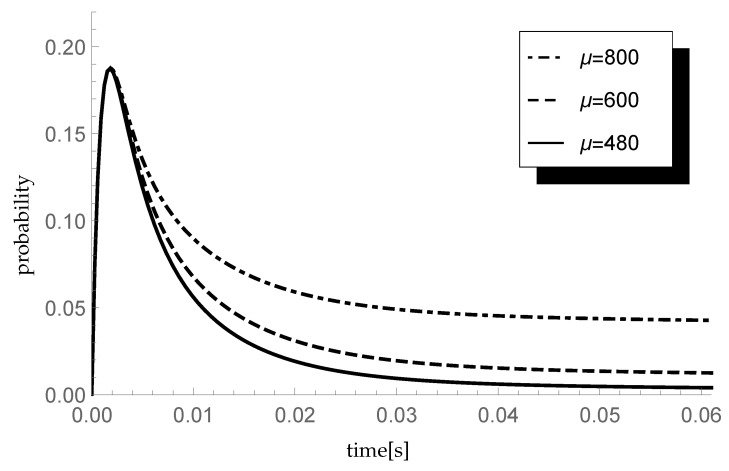
Impact of service rate on probability P{X(t)=1|X(0)=0}.

Hence, μ is equal to 800, 600 and 480 packets/s that correspond to processing rates 640, 480 and 384 kb/s, respectively. Keeping constant the arrival intensity λ=600 packets/s, we have the traffic load of the system ϱ=0.75,1 and 1.25, respectively. Assume that the distribution of the arriving group size is given by the sequence $p_{k}=\left\{0.5,0.5,0,\ldots \right\}$ so the mean group size equals 1.5. Moreover, let ξ=1000 so the mean single vacation duration equals 0.002 s. In Figure 1, transient conditional distributions P{X(t)=1|X(0)=0} are visualized.

Observe that the probability P{X(t)=1|X(0)=0} is the highest just after the opening of the system. This is due to the fact that, after starting, the server immediately goes into the vacation mode due to the lack of packets to handle.

### 5.2. Impact of Arrival Rate

**Figure 2:**
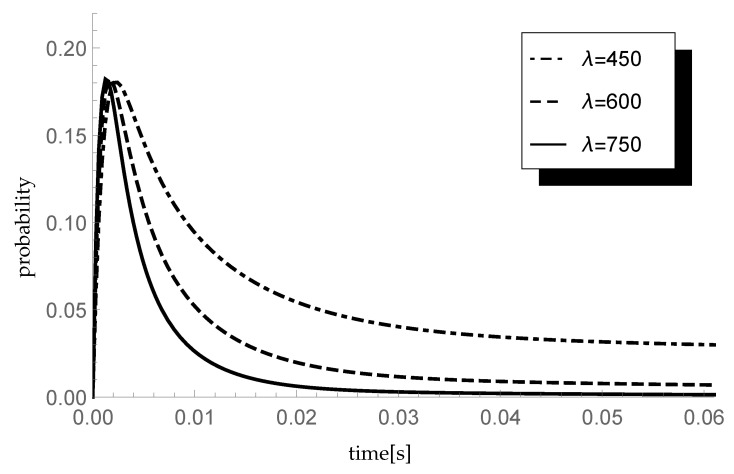
Impact of arrival rate on probability P{X(t)=1|X(0)=0}.

Analyze now the impact of the arrival rate λ on the transient queue-size distribution. Consider three scenarios in which λ=450,600 and 750 packets/s, which corresponds to data transfer rates of 360,480 and 600 kb/s. The parameters of the CDF of the processing time are $\alpha =0.6,\mu_{1}=680$ and $\mu_{2}=510$, which gives the mean service rate μ=600. The values of the traffic load for successive scenarios are 0.75,1 and 1.25, respectively. Moreover, as previously, ξ=1000 so the average single vacation duration equals 0.002 s. Taking $\left(p_{k}\right)=\left\{0.5,0.25,0.25,0,\ldots \right\}$, in Figure 2 probabilities P{X(t)=1|X(0)=0} are presented.

As time passes, the probability decreases until the system reaches equilibrium. The nature of the decreasing probability depends on the arrival intensity: the higher the λ, the faster the system reaches equilibrium.

### 5.3. Impact of Initial Buffer State

**Figure 3:**
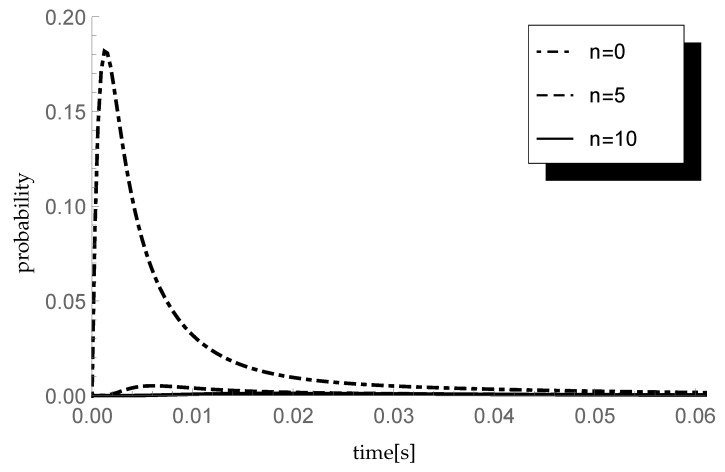
Impact of initial buffer state on probability P{X(t)=1|X(0)=0} for different $n^{\prime }s.$

Investigate now the impact of the initial buffer state *n* on the transient probability P{X(t)=1|X(0)=n} for n=0,5 and 10. Assume λ=450 packets/s, which corresponds to the arrival rate 600 kb/s. Moreover, take the service rate μ=600 packets/s so the offered traffic load ϱ equals 0.75. Taking the same parameter ξ=1000 of the single vacation duration CDF and the sequence $\left(p_{k}\right)=\left\{0.5,0.25,0.25,0,\ldots \right\}$ describing sizes of arriving groups, the transient behavior of the considered probability is visualized in Figure 3.

As can be seen, for small values of time *t*, the influence of the initial buffer state is clearly visible and the differences between corresponding probabilities are relatively large.

### 5.4. Impact of Single Vacation Duration

**Figure 4:**
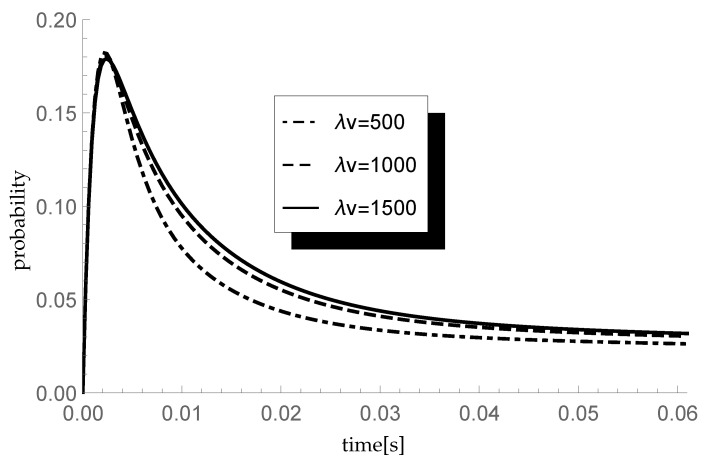
Impact of single vacation duration on probability P{X(t)=1|X(0)=0} for λ=450 packets/s.

**Figure 5:**
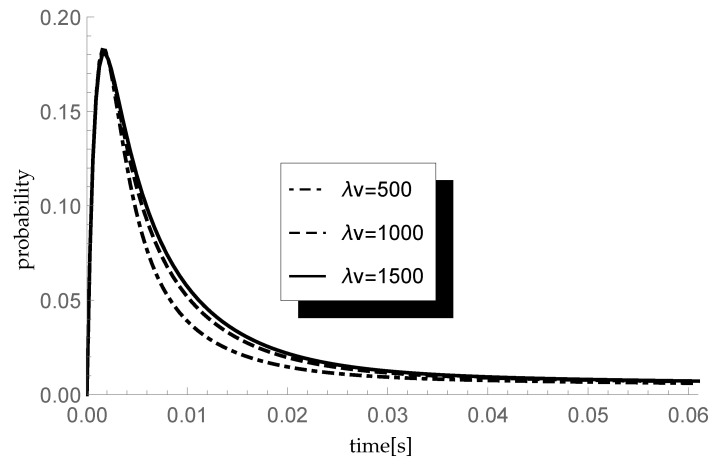
Impact of single vacation duration on probability P{X(t)=1|X(0)=0} for λ=600 packets/s.

**Figure 6:**
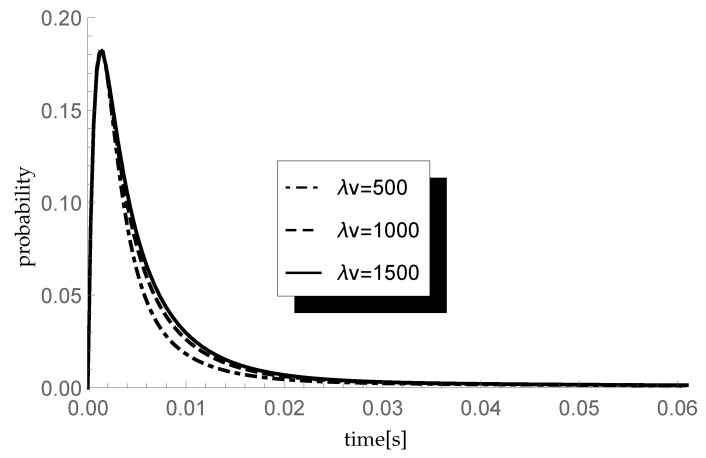
Impact of single vacation duration on probability P{X(t)=1|X(0)=0} for λ=750 packets/s.

Finally, let us check the response of the transient queue-size distribution to changes in the average length of a single vacation. Consider three different scenarios in which the arrival intensity λ takes, as previously, values 450, 600 and 750 packets/s and the service rate μ=600 packets/s. In consequence, ϱ=0.75,1 and 1.25, respectively. In each scenario we analyze the behavior of the transient probability P{X(t)=1|X(0)=0} for three different values of parameter ξ=λv of the single vacation duration CDF, namely 500,1000 and 1500 (corresponding to mean single vacation durations 0.004, 0.002 and 0.0013 s). The size of an arriving group of packets is distributed according to sequence $\left(p_{k}\right)=\left\{0.5,0.25,0.25,0,\ldots \right\}$ so the mean group size equals 1.75. Results of the experiment are presented in Figure 4, Figure 5 and Figure 6.

Let us comment briefly on the final results. As one can observe, the longer the mean single vacation duration (the smaller the value of parameter ξ=λv), the smaller the probability P{X(t)=1|X(0)=0}. Indeed, longer vacations are conducive to the accumulation of more packets in the buffer before the start of the processing. This phenomenon is particularly visible at the lowest traffic load value ϱ=0.75.

### 5.5. Numerical Computations vs. Simulation Results

In this subsection, we compare numerical results with the simulation model created in Objective Modular Network Testbed in C++ (OMNeT++) [45]. We modify the standard FIFO model imbedded in OMNeT++ discrete event simulator in a way to transmit and queue batches of packets according to the following scenarios:

- The transient probability P{X(t)=1|X(0)=0} is calculated for λ=μ=600 packets/s (resulting in ϱ=1). We take ξ=1000 as a parameter of the single vacation duration CDF (corresponding to mean single vacation duration 0.002 s) and the sequence $\left(p_{k}\right)=\left\{0.5,0.25,0.25,0,\ldots \right\}$ describing sizes of arriving groups (so the mean group size equals 1.75);
- As in the previous scenario except for the sequence $\left(p_{k}\right)=\left\{1,0,0,\ldots \right\}$ (single arrivals).

The simulation model is extended by a time step of $t_{c}=0.0005$ s, describing the time moments $k\cdot t_{c}=t\in \left[0,0.06\right]$ for k=0,1,2,…,60, at which the number of packets present in the system is measured. The simulation run is divided into 10,000 individual trials. Each attempt is a separate time period t∈[0,0.06] in which the random processing is simulated in accordance with the multiple vacation policy assumed in our queueing model. A unique seed is randomly selected for each sample and used to initialize the pseudorandom number generator representing random arrival and service as well as the lengths of individual vacation periods.

**Figure 7:**
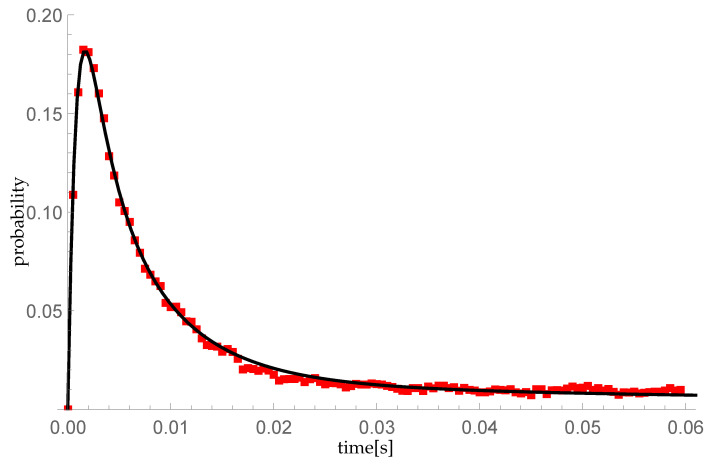
Comparison of the P{Y(t)=1|Y(0)=0} probability distribution for the compound Poisson process, obtained: (1) by numerical calculations using the formula (28)—black line on the graph; (2) as a statistical result of a 10,000th random sample—the red squares in the diagram.

**Figure 8:**
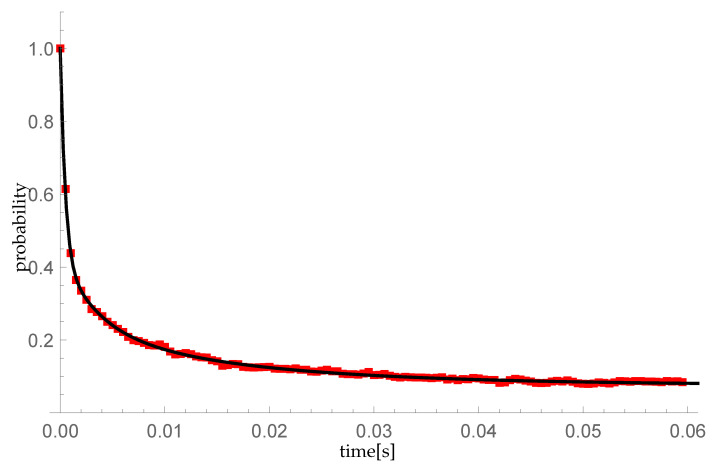
Comparison of the P{Y(t)=1|Y(0)=0} probability distribution for the simple Poisson process, obtained: (1) by numerical calculations using the formula (28)—black line on the graph; (2) as a statistical result of a 10,000th random sample—the red squares in the diagram.

Figure 7 and Figure 8 confirm the compliance of the numerical calculations with the simulation results. The maximum difference in the probability values between the numerical calculations and the simulation results measured at times $k\cdot t_{c}$ equals 0.00599106.

## 6. Conclusions

In this article, transient queue-size distribution in the batch-arrival system with multiple vacation policy is studied. For the purposes of the analysis, the approach based on the idea of the embedded Markov chain, continuous formula of total probability, integral equations and linear algebra is proposed. As the main result, the closed-form formula for the Laplace transform of the transient queue-size distribution conditioned by the initial buffer state is obtained. In the numerical study, the impact of the service rate, arrival intensity, initial buffer state and mean single vacation duration on the studied characteristic is investigated for examples of the considered queueing model. The considered queueing model has many potential applications in modeling the operation of production systems, computer and telecommunication networks with an energy saving mechanism based on cyclic monitoring the state of the queue of packets (messages, tasks, etc.) waiting for service. During the multiple vacation period, the service station may perform other types of tasks, such as, for example, the time the CPU is running in the background, installing software updates or maintaining the server. In applications in modeling the operation of an automated production line, during downtime, a single machine (service station) can be used in secondary production; during this time, the machine can also be calibrated or the settings can be changed. Due to the fact that, in some practical applications, interarrival times are not exponentially distributed, it is planned in future to extend the analysis for the case of a non-Poisson arrival stream (e.g., hyper-exponential or a general renewal one).

## Data Availability

Data are available upon reasonable request. Additional data (beyond those included in the main text are available from the corresponding author upon request.
